# Genetic Analysis of the Capsular Biosynthetic Locus from All 90 Pneumococcal Serotypes

**DOI:** 10.1371/journal.pgen.0020031

**Published:** 2006-03-10

**Authors:** Stephen D Bentley, David M Aanensen, Angeliki Mavroidi, David Saunders, Ester Rabbinowitsch, Matthew Collins, Kathy Donohoe, David Harris, Lee Murphy, Michael A Quail, Gabby Samuel, Ian C Skovsted, Margit Staum Kaltoft, Bart Barrell, Peter R Reeves, Julian Parkhill, Brian G Spratt

**Affiliations:** 1 Sanger Institute, Wellcome Trust Genome Campus, Hinxton, Cambridge, United Kingdom; 2 Department of Infectious Disease Epidemiology, Imperial College, London, United Kingdom; 3 School of Molecular and Microbial Biosciences, University of Sydney, Sydney, Australia; 4 Staten Serum Institut, Copenhagen, Denmark; The Institute for Genomic Research, United States of America

## Abstract

Several major invasive bacterial pathogens are encapsulated. Expression of a polysaccharide capsule is essential for survival in the blood, and thus for virulence, but also is a target for host antibodies and the basis for effective vaccines. Encapsulated species typically exhibit antigenic variation and express one of a number of immunochemically distinct capsular polysaccharides that define serotypes. We provide the sequences of the capsular biosynthetic genes of all 90 serotypes of Streptococcus pneumoniae and relate these to the known polysaccharide structures and patterns of immunological reactivity of typing sera, thereby providing the most complete understanding of the genetics and origins of bacterial polysaccharide diversity, laying the foundations for molecular serotyping. This is the first time, to our knowledge, that a complete repertoire of capsular biosynthetic genes has been available, enabling a holistic analysis of a bacterial polysaccharide biosynthesis system. Remarkably, the total size of alternative coding DNA at this one locus exceeds 1.8 Mbp, almost equivalent to the entire S. pneumoniae chromosomal complement.

## Synopsis

Several major bacterial pathogens are coated by a polysaccharide capsule that is important for virulence. Each strain of Streptococcus pneumoniae (the pneumococcus) produces one of 90 different capsular polysaccharides, which are distinguished by using a set of antisera that recognise the chemical differences in the capsules. The capsule is important for virulence, but is immunogenic, and the large number of different capsular serotypes is believed to have been selected as a mechanism to evade the human immune response. Antibodies against capsular polysaccharide can protect against pneumococcal disease, and a highly effective protein-conjugated polysaccharide vaccine that protects children against the seven serotypes most commonly associated with serious disease has recently been introduced in the United States. Bentley and colleagues have determined the DNA sequence of the capsular biosynthesis genes for all 90 serotypes of S. pneumoniae and, by correlating the gene content with the serological profiles and with the known capsular polysaccharide structures, provide what they believe to be the most detailed and complete understanding of capsular biosynthesis and capsular gene evolution in any bacterial pathogen.

## Introduction

Streptococcus pneumoniae (the pneumococcus) is a major cause of morbidity and mortality worldwide, causing diseases that range in severity from meningitis, septicaemia, and pneumonia to sinusitis and acute otitis media [1,2]. Factor (typing) sera are used to divide pneumococci into serotypes and serogroups, which include immunologically related serotypes. These sera have been developed by a process of multiple cross-absorptions, which render them specific for the immunochemical differences between the pneumococcal capsular polysaccharides (CPSs) [3]. At present, 90 individual serotypes are recognised by their patterns of reactivity with the factor sera [4], and serotypes vary in the extent to which they are carried in the nasopharynx and the degree to which they are recovered from different disease states [5,6]. Expression of a capsule is important for survival in the blood and is strongly associated with the ability of pneumococci to cause invasive disease. The capsule is surface exposed, and antibodies against CPS provide protection against pneumococcal disease. Consequently, polyvalent polysaccharide vaccines have been developed in which CPS from the serotypes most commonly associated with invasive disease in children are linked to a protein carrier, and a seven-valent conjugated polysaccharide vaccine has been introduced and shown to be highly effective [7,8]. A 23-valent polysaccharide vaccine is also available for use in adults [9].

**Figure 1:**
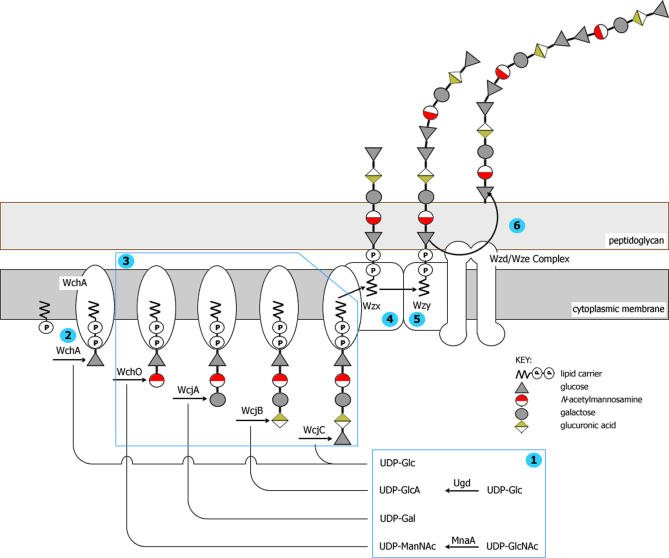
Representation of the Wzx/Wzy-Dependent Pathway for Biosynthesis of CPS 9A Pictured is a hypothetical model for capsule biosynthesis in S. pneumoniae based on a mixture of experimental evidence and speculation. For a recent review, see Yother [15]. (1) Non-housekeeping nucleotide sugar biosynthesis. (2) The initial transferase (WchA in this case) links the initial sugar as a sugar phosphate (Glc-P) to a membrane-associated lipid carrier (widely assumed to be undecaprenyl phosphate). (3) Glycosyl transferases sequentially link further sugars to generate repeat unit. (4) Wzx flippase transports the repeat unit across the cytoplasmic membrane. (5) Wzy polymerase links individual repeat units to form lipid-linked CPS. (6) Wzd/Wze complex translocates mature CPS to the cell surface and may be responsible for the attachment to peptidoglycan. The complex of WchA, Wzy, Wzx, Wzd, and Wze shown in the membrane is based on that in Figure 2 of Whitfield and Paiment [47] for the related Escherichia coli Type 1 capsule.

With the exception of types 3 and 37, which are synthesised by the synthase pathway [10–14], pneumococcal CPSs are generally synthesised by the Wzx/Wzy-dependent pathway (Figure 1). The genes for the latter pathway are located at the same chromosomal locus *(cps),* between *dexB* and *aliA* [15–17]. CPSs are synthesised by transfer of an initial monosaccharide phosphate from a nucleotide diphosphate sugar to a membrane-associated lipid carrier, followed by the sequential transfer of further monosaccharides to produce the lipid-linked repeat unit. This is transferred to the outer face of the cytoplasmic membrane by the repeat-unit transporter or flippase, polymerised to form the mature CPS, and then attached to the peptidoglycan [18]. The *cps* locus therefore typically encodes the enzymes to build the repeat unit, including an initial glycosyl phosphate transferase, and additional transferases responsible for the formation of the linkages, and to allow for the addition of sugars (or other moieties), or to otherwise modify the repeat unit, as well as a repeat-unit flippase and polymerase [15].

**Figure 2:**
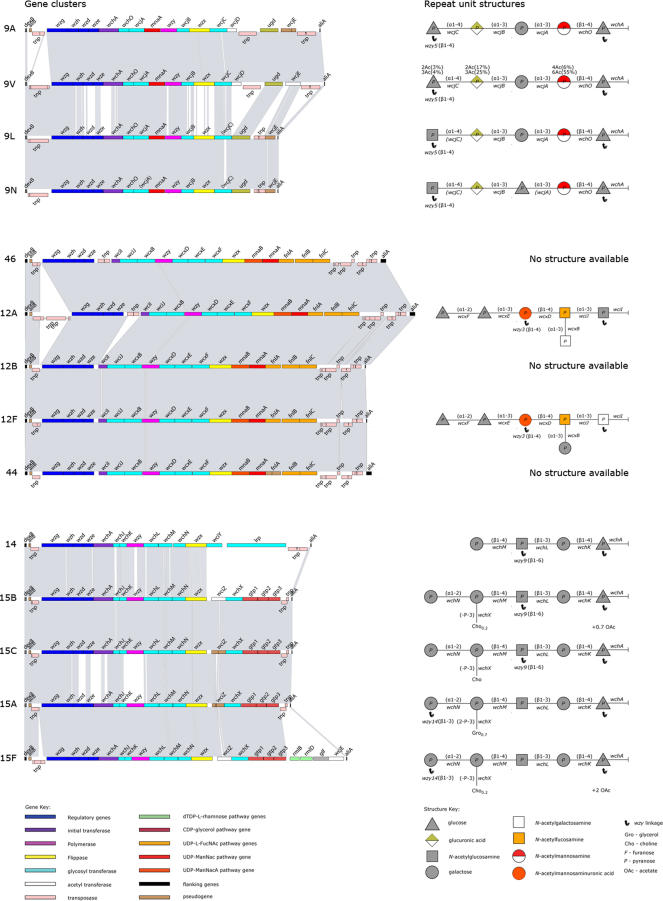
Capsule Biosynthesis Genes and Repeat-Unit Polysaccharide Structures Shown are the *cps* gene clusters for cases discussed in the text, together with the polysaccharide structure of the encoded repeat unit where known [31] (the full set is shown in Figure S1). Genes are represented on the forward and reverse strands by boxes coloured according to the gene key, with gene designations indicated above each box. Grey blocks indicate regions of sequence similarity between gene clusters. Repeat-unit structures are displayed with the linkage to undecaprenyl pyrophosphate at the right-hand side (not necessarily the case for the published structures [31]), so residue numbers are counted from right to left. Monosaccharides are represented as shapes coloured according to the structure key. Housekeeping sugars are coloured grey. Non-housekeeping sugar colours correspond to the associated sugar biosynthesis gene colours. Glycerol, choline, and acetate are indicated as text. Also shown are the nature of linkages with the associated gene, and the linkages between repeat units created by the Wzy polymerase. Gene designations are in parentheses where their substrate specificity is unclear.

The substantial diversity of pneumococcal CPSs is believed to have arisen as a consequence of selection for antigenic diversity imposed by the human immune system [6]. The evolutionary timescales and the genetic events by which novel serogroups and serotypes arise are unclear. Comparisons of the available *cps* loci indicate a variety of genetic mechanisms and show that the central genes responsible for the synthesis and polymerisation of the repeat unit are highly variable and often non-homologous between serotypes. These genes have a low percentage G+C content, and new serotypes may frequently have been generated by the introduction of novel *cps* genes into pneumococci by lateral gene transfer from other species. A much better understanding of the complex mechanisms by which antigenic diversity arises could be obtained by using the sequences of the complete set of pneumococcal *cps* loci. We therefore obtained sequences of the *cps* locus for all 90 serotypes and used these data, together with the available polysaccharide structures and the patterns of serological reactions with typing sera, to explore the genetics of capsular diversity in this major pathogen. Here we present highlights of our analysis to date, and a more exhaustive analysis will be reported elsewhere.

## Results

### General Features of the *dexB–aliA* Locus from 90 Serotypes

PCR products were generated from genomic DNA using primers specific for the *dexB* and *aliA* genes and ranged in size from 10,337 bp (serotype 3) to 30,298 bp (serotype 38) with an average of 20,714 bp. The synthase gene *(wchE)* of serotype 3 is located within the *cps* locus, but the type 37 *cps* locus, which was very similar to that of serotype 33F, is defective and serotype is determined by the type 37 synthase gene *(tts)* located elsewhere on the chromosome [10]. Annotation and analysis of the *cps* sequences revealed the generality of several previously observed characteristics. Genes for the generation of CPSs are always orientated in the same direction as the *dexB* and *aliA* genes (Figures 2 and S1). The regulatory and processing genes *wzg, wzh, wzd,* and *wze* (also known as *cpsABCD*) are conserved with high sequence identity in all cases and are almost always in this gene order at the 5′ end of the *cps* locus. In most *cps* clusters, the fifth gene encodes the initial glucose phosphate transferase, WchA (also known as CpsE), responsible for linkage of an activated glucose phosphate to the lipid carrier (see below). The polysaccharide polymerase *(wzy)* and flippase *(wzx)* genes are always present downstream together with a varying set of genes for glycosyl transferases, acetyl transferases, nucleotide diphosphate sugar biosynthesis, and modifying enzymes.

In every case, there is a region of low percentage G+C content within the *cps* locus. The first four genes and the non-housekeeping sugar biosynthesis genes have typical percentage G+C content for *S. pneumoniae,* while the “serotype-specific” genes, particularly *wzy* and *wzx,* tend to have more AT-rich sequences. In the regions between the *cps* genes and the flanking *dexB* and *aliA* genes, there is almost always evidence of mobile genetic elements. This is largely manifested as intact or disrupted genes for insertion-sequence (IS) transposases [19,20], although in four cases we identified group-II introns [21] (serotypes 19F, 25F, 25A, and 38).

We could assign a functional designation to the products of all but 26 of the 1,999 predicted coding sequences in the 90 *cps* regions, with most of the remainder showing weak similarities to products of genes in bacterial polysaccharide gene clusters. Unsurprisingly, many coding sequences fall into the broad functional categories of glycosyl transferase (351), acetyl transferase (74), and sugar phosphate transferase (71). To make more specific assignments within such categories, we used the TribeMCL program to assemble all the annotated proteins into homology groups (HGs). With from two to 90 members in each, 91% of the proteins assembled into 175 HGs, with the remainder forming 74 single-member HGs (Table S1). The products of *wzg, wzh, wzd,* and *wze* each fall into a single HG covering every serotype. Ignoring IS element transposases, the next largest HG comprises 65 WchA initial transferases (HG5). At the other extreme, the serotype-specific gene products are diverse, with 87 HGs for non-initial sugar transferases and 40 and 13 groups of Wzy repeat-unit polymerases and Wzx flippases, respectively.

### Biosynthesis of Precursors for Sugars and Other CPS Components

Of the 18 sugars and related compounds found in S. pneumoniae capsules, seven are available from housekeeping metabolic pathways and nine from known dedicated pathways encoded within the *cps* cluster (Figure S2). This includes 4-keto-*N*-acetyl-D-quinovosamine (UDP-KDQNAc), which is the intermediate in the two step reaction catalysed by FnlA [22]. We found a perfect correlation between the presence of a non-housekeeping sugar in the CPS and the presence of the appropriate biosynthetic genes in the *cps* locus. Two of the three remaining compounds are the sugar alcohol phosphates arabinitol-1-P and mannitol-6-P. The precursors for these have not been identified, but nucleotide-diphosphate-linked precursors can be easily derived from D-xylulose-5-phosphate or D-fructose-6-phosphate, respectively, by two-step pathways parallel to that for CDP-ribitol formation from ribulose-5-phosphate [23]. D-xylulose-5-phosphate and D-fructose-6-phosphate are central to major pathways, and there are appropriate genes for their conversion in the associated *cps* loci. The precursor for ribofuranose has also not been identified, but a proposed pathway for its biosynthesis by the product of a gene within CPS 19F *(cps19R)* [24] is supported by our observation that an orthologous gene (renamed *rbsF*) is present for all CPS that contain ribofuranose.

Choline-1-phosphate, glycerol-1-phosphate, and glycerol-2-phosphate are also found in some of the structures. CDP-choline is known to be produced by S. pneumoniae as a precursor for teichoic acid biosynthesis [25]. For glycerol-1-phosphate, we find an intact *gct* gene for CDP-glycerol synthesis [26] in the *cps* where expected, and there are four genes associated with presence of glycerol-2-phosphate, three of which are thought to encode a CDP-2-glycerol pathway [27], while *wchX* encodes the glycerol phosphotransferase.

The situation is illustrated in Figure 1 for *cps*9A, which has pathway genes for *N*-acetylmannosamine pyranose and glucuronic acid, but not for glucopyranose (Glc*p*) or galactopyranose (Gal*p*) as these are available in S. pneumoniae from central metabolism.

### Initial Transferases and Polymerisation

Initial transferase WchA adds glucose-1-phosphate to undecaprenol phosphate [28] to create Und-PP-Glc (Figure 1), and we assume it performs that function in all 65 serotypes where it is present. For the known structures, there is a perfect correlation between the presence/absence of *wchA* and the presence/absence of glucose in the repeating unit. Where *wchA* is absent, the products of the fifth *cps* gene fall into three HGs (WciI, WcjG, and WcjH) all with the same Pfam [29] domain and similar hydrophobicity profiles to the carboxy-terminal region of WchA. We suggest that they function as the initial sugar transferases, as it is known that for the *Salmonella enterica wchA* homologue, *wbaP,* the 3′ end of the gene is sufficient for transferase activity [30]. By correlation with CPS constituents, we predict the transferred initial sugars as *N*-acetylgalactosamine pyranose (Gal*p*NAc) or *N*-acetylglucosamine pyranose (Glc*p*NAc) for WciI, Gal*p* or galactofuranose for WcjG and Gal*p* for WcjH. Serotype 1 is an exception as no gene product with similarity to an initial sugar transferase has yet been identified.

The initial sugar of the repeat unit is also the donor sugar in the polymerisation of the repeat units (Figure 1), and the specificity of the Wzy polymerase determines the other component of this linkage, which in the case of CPS 9A is a beta (1–4) linkage to the terminal glucose of the next repeat unit. For the known structures [31], identification of the initial sugar allowed us to determine the polymerase linkage as both donor and acceptor sugar, and the linkages were defined once the initial sugar had been identified (see Figures 2 and S1). Where there is ambiguity due to two residues of the initial sugar in the repeat unit, the polymerase linkage can be provisionally identified by considering the linkage catalysed by other members of the same Wzy HG. The predictions for initial sugars, and subsequent repeat-unit polymerisation linkage, correlate well with the polymerase HGs (Table S2). There are 32 polymerase HGs associated with WchA, five with WciI, four with WcjG and one with WcjH. These associations are mostly exclusive, with only five polymerase HGs associated with two initial transferases. In such cases, the linkages involve the same acceptor sugar anomerism (α or β isomer) and the same or a closely related donor sugar. This adds strong support to the inferences drawn for the specificity of the initial transferases.

### Relating *cps* Genes with CPS Structure and Serological Profile

The availability of all of the annotated *cps* sequences allowed us to look for correlations between genes, known CPS structures, and serology (gene clusters, CPS structures, and antigenic formulae are summarised in Figure S1 and Table S3). In this way, we can attempt both to infer gene function and, by comparing related *cps* loci, to account for differences in CPS structure and serology. Variations between *cps* loci range from two base substitutions for 18B and 18C to wholesale differences in gene complement. Within this range, the variations likely to have a phenotypic effect include gene inactivation due to single base substitutions generating a premature stop codon, single base insertion/deletions leading to translational frameshifts, change of sequence leading to change of enzyme specificity, recombination or IS element insertion leading to gene truncation, and insertion/deletion/replacement of single and multiple genes. Within serogroups, the genetic differences were often subtle but were also sometimes surprisingly prominent. Comparisons also revealed some strong commonality between the *cps* of different serogroups and serotypes. Illustrative examples that demonstrate how structure, genetics, and serology were combined to analyse the *cps* loci are shown in Figure 2 and are discussed below.

### Serogroup 9

Previously described CPS structures [31] for all four serotypes of serogroup 9 show only subtle differences and provide an example of multiple serotypes arising by divergence from a single *cps* locus. Their *cps* genes fall into two pairs, with 9A highly similar to 9V [32], and 9L highly similar to 9N, but with the two pairs differing significantly in sequence ( Figure 2), suggesting an initial divergence to form two ancestral serotypes; this split correlates with a difference at residue 5 of the repeat unit, where 9L and 9N CPSs have Glc*p*NAc, whereas 9A and 9V have Glc*p*. Factor sera 9d reacts with 9A and 9V but not with 9L and 9N, suggesting that it is interacting with Glc*p* but not with Glc*p*NAc. Both are housekeeping sugars, and their differential incorporation is likely to be due to divergent forms of glycosyl transferase WcjC. Subsequently, one of these ancestral serotypes diverged to form 9L and 9N, the latter becoming unique in the group in having Glc*p* rather than Gal*p* as residue 3 in the repeat unit. Their *dexB–aliA* loci have the same gene complement, and within the *cps* genes there are only 79 nucleotide differences. The highest number of amino acid substitutions (13) is within glycosyl transferase WcjA; ten are unique to 9N and presumably result in its altered specificity for Glc*p* rather than for Gal*p*.

The other ancestral serotype gave rise to 9V and 9A, which differ from each other only in their CPS acetylation; the former CPS has an *O*-acetylation pattern unique in the serogroup. This is likely due to the *O*-acetyl transferase–encoding *wcjE* gene, which is intact and apparently functional in 9V, disrupted by a frameshift mutation in 9A (deletion of guanine, nucleotide 726), and truncated in 9L and 9N by the insertion of an IS element. Interestingly, factor sera 9g reacts only with serotype 9V and may recognise an acetyl-based epitope determined by *wcjE*.

Serogroup 9 *cps* loci also differ by the insertion, in 9A and 9V relative to 9L and 9N, of an *O*-acetyl transferase gene *(wcjD)* and an adjacent IS element. This correlates with recent nuclear magnetic resonance data (I. C. Skovsted, unpublished data), indicating that 9A CPS is partially acetylated.

### Serotypes 44 and 46 Are Related to Serogroup 12

The *cps* gene clusters of serogroup 12 and serotypes 44 and 46 are almost identical, differing only in IS transposase genes, and provide an example of common ancestry that is not apparent from serology. Structures have been determined for serotypes 12F and 12A only, although the individual constituents for serotype-46 CPS are known and all are present in 12F and 12A [31]. Although no factor serum cross-reacts with all five serotypes, serological reactions do indicate antigenic commonalities [4]; 44 cross-reacts with factor sera 12b and 12d, while 46 cross-reacts with 12c. Given the *cps* similarities, the significant differences between 12F and 12A CPS are perhaps surprising; 12A has a Gal*p*NAc and 12F has a Gal*p* side branch, and the first main-chain residue is Gal*p*NAc in 12F and Glc*p*NAc in 12A. The nucleotide differences are concentrated within two glycosyl transferase genes *(wciI* and *wcxB),* and we predict that the initial transferases, WciI–12A and WciI–12F, with 38 amino acid differences, link Glc*p*NAc and Gal*p*NAc, respectively, to the lipid carrier, while WcxB–12A and WcxB–12F, with 17 amino acid differences, account for the side-branch difference.

### Serotype 14 Is Closely Related to Serogroup 15

Serotype 14 shares no significant serological cross-reaction with serogroup 15, or with any other serotype, but the *cps* loci of these two serotypes are clearly related. All CPS structures for serotype 14 and serogroup 15 are known [31,33,34], and comparisons of structures and genes allow inferences about one to be made from the other. The four serogroup 15 pentasaccharide repeat units are identical, but polymerisation forms a linear polymer in 15A and 15F and a branched structure in 15B and 15C that correlates with the presence of *wzy* genes of different HGs (see Table S2). Serotypes 15B and 15C differ in the presence or absence of *O*-acetylation [35] and, as previously described [36], the difference is due to a variable-length TA tandem repeat region at the 5′ end of *wciZ*—in frame in 15B and out of frame in 15C strains. This gene is in frame in 15F (acetylated), but extensively degraded, rather than simply out of frame, in 15A (not acetylated).

Genes for synthesis of glycerol-2-phosphate *(gtp1, gtp2,* and *gtp3)* are present in all serogroup 15 *cps* loci, but glycerol was reported to be present only in 15A, being replaced by choline-P in 15F, 15B, and 15C, with either residue being present on only a proportion of the repeat units [31]. In all cases, the transferase is presumed to be encoded by *wchX*, with the molecular basis of the structural polymorphism being contentious. However, recent nuclear magnetic resonance analysis indicates that 15B contains glycerol and not choline, suggesting that the same may also be true for 15F and 15C [34]. The 3′ end of 15F *cps* has four extra genes—*rmlB, rmlD, glf,* and a putative acetyl transferase gene *wcjE*—but they appear to have no effect on the structure as there is no rhamnose, galactofuranose, or extra acetylation in 15F CPS. Indeed, *rmlA* and *rmlC* would also be required for rhamnose biosynthesis. These four genes show synteny with the 3′ end of *cps* in several serotypes, particularly serotype 31, and their arrangement in 15F may indicate a recombination event.

The serotype 14 [28,37,38] and basic 15 *cps* gene clusters clearly share common ancestry and differ only at the 3′ end, where the glycerol-2-phosphate–related genes in 15 are replaced in 14 by a gene *(lrp)* encoding a large (1,359 amino acid) repetitive protein, which correlates well with CPS structures [36]. The type-14 repeat unit most resembles the branched form of 15B and 15C, with the lack of *O*-acetylation due to the absence of *wciZ*. The lack of α-D-galactose is probably due to degradation of the relevant transferase gene, *wchN*. The large repetitive protein encoded by serotype-14 *cps* has a hydrophobic C-terminal region, suggesting that it may be anchored to the cell surface. This leads us to speculate that Lrp may serve as a dominant antigen that overwhelms the serological similarities to serogroup 15 that should be evident from their very similar repeat units.

## Discussion

Several bacterial pathogens exist as a large number of antigenic variants because of differences in the polysaccharides presented at the cell surface. However, the sequencing and analysis of the *cps* loci of pneumococci described here are believed to provide the only such case where the whole gene repertoire is available, allowing genetics, chemistry, and immunology to be combined to predict the role of *cps* genes. This combined approach has allowed the confident prediction of most gene functions, but it has also highlighted the limitations where subtle sequence changes may alter enzyme substrate specificity. Analysis of the *cps* loci indicates that a number of different mechanisms have generated antigenic diversity in CPSs. Some of these involve the divergence of a single serotype into two related serotypes by the accumulation of point mutations (e.g., serogroup 6 [39]), or the insertion or deletion of a single gene, resulting in slightly different CPS structures (e.g., serogroup 18). In other cases, the *cps* loci of some serotypes within a serogroup seem to be virtually unrelated and probably reflect the sharing of a dominant epitope that led to them being placed within the same serogroup (e.g., serogroups 7, 17, 33, and 35). Similarly, some serotypes placed in different serogroups show more relatedness among their *cps* loci than those within the same serogroup (e.g., types 7B and 7C are more closely related to type 40 than to 7A and 7F). This is perhaps not surprising as serogroups were defined by common epitopes in the absence of any knowledge of the CPS structures or the *cps* sequences that code for their synthesis. Shared immunodominant epitopes will lead to inclusion in the same serogroup even if there are major differences in other parts of the structure and hence in the *cps*.

A striking feature of the *cps* loci is the presence of many highly divergent forms of each of the key enzyme classes. Thus, there are 40 HGs for polysaccharide polymerases, 13 groups of flippases, and a great diversity of transferases. The presence of multiple non-homologous or highly divergent forms of these enzymes, together with the low percentage G+C content of the region in which these are encoded, supports the view that these genes have been imported into pneumococci (or their ancestors) on multiple occasions from different and unknown sources. The plethora of transferases in the pneumococcal *cps* loci provides an opportunity to continually generate new serotypes by gene shuffling, but there are no clear examples of serotypes arising as mosaics of two existing *cps* loci. One barrier to the frequent appearance of new serotypes by recombination is a lack of homology between the serotype-specific regions of *cps* loci of different serogroups. The appearance of new serotypes may also be limited by a need to change multiple *cps* genes; rare genetic events that create mosaics between existing *cps* loci probably typically fail to produce a capsule since new repeat units resulting from the capture of novel transferases are unlikely to be recognised as substrates by the resident repeat-unit polymerase. The *cps* sequences, and their associated polysaccharide structures and serological profiles, constitute an extensive dataset that, through further detailed analysis, will allow a clearer understanding of capsule biochemistry, genetics, and evolution and will precipitate advances in molecular serotyping of pneumococci [40,41].

## Materials and Methods

### Strain selection, serotyping, and genomic DNA isolation.

Representative strains of the 90 S. pneumoniae serotypes were selected from among the lyophilised strains in the strain collection of the World Health Organization Collaborating Centre for Reference and Research on Pneumococci, Statens Serum Institut (Copenhagen, Denmark) (Table S4). The strains were serotyped and cultured, and genomic DNA was extracted by standard methods [3,4,42].

### PCR and DNA sequencing.

PCR reactions were performed using the Expand Long Template PCR System (Roche, Basel, Switzerland), which contains proof-reading thermostable polymerases. Initial reactions used primers CPS1 (TTGCCAATGAAGAGCAAGACTTGACAGTAG) and CPS2 (CAATAATGTCACGCCCGCAAGGGCAAGT) [26]. Where these failed to produce an adequate product, further reactions were attempted using alternative *dexB*-specific primers (CPS1A [CGACCGTCGCTTCCTAGTTGTGGCTAAC] or PCPS3f [CACACAGAAAGCATCCCATGG]) and *aliA*-specific primers (CPS1B [GTCTTGAGCTTTGACTGCCGCGTATTCT] or PCPS3r [GAGACAGACCTGATAACCTCAACTATTTG]). The *cps* cluster for our serotype-5 strain was amplified using a primer based on the EMBL file (AY336008) specific for the *wzg* gene (CPS05F [CGTTCACAGAAAGTGAAGCG]) in combination with PCPS3r. PCR products spanning the *cps* locus were used directly to construct small-insert libraries [43], with 1- to 2-kb inserts in pUC18. Clones from each library were sequenced from each end using Big-Dye terminator chemistry (Applied Biosystems, Foster City, California, United States) on ABI3730 sequencing machines, to give an average of 8- to 10-fold coverage of each product. These reads were assembled with Phrap (CodonCode, Dedham, Massachusetts, United States), and any gaps or regions of poor coverage were re-sequenced using primer-directed sequencing directly from the original PCR product using Big-Dye primer chemistry (Applied Biosystems). This sequencing procedure should prevent any PCR errors from being represented in the final consensus sequence.

### Annotation and bioinformatic methods.

Gene prediction and annotation were performed as previously described [44]. Predicted proteins were clustered into homology groups using TribeMCL (Centre for Mathematics and Computer Science and EMBL-EBI) [45] with a cut-off of 1*e* ^−50^. The genes within the *cps* loci that encoded proteins within the same homology group were assigned the same name, the exceptions being the polymerases and flippases where we used the prior gene nomenclature, *wzy* and *wzx,* even though in both cases there were multiple homology groups. Alignment of gene clusters was performed using the Artemis Comparison Tool (Sanger Institute, Hinxton, United Kingdom). Nucleotide differences were identified using the EMBOSS program Diffseq (MRC Rosalind Franklin Centre for Genomics Research, Hinxton, United Kingdom) [46].

## Supporting Information

Figure S1 Capsule Biosynthesis Genes and Repeat-Unit Polysaccharide Structure for All 90 Serotypes (9.9 MB TIF)

Figure S2 Biosynthesis Pathways for Non-Housekeeping Sugars (50 KB PPT)

Table S1 Homology Groups including Numbers of Members and Product Description Proteins in different homology groups are so divergent that they are highly unlikely to have diverged from a common streptococcal ancestor.
(306 KB DOC)

Table S2 Associations between Initial Transferases and Wzy Polymerase Groups Proposed Wzy groupings represent a sequential numbering of homology groups and are represented on structural diagrams.
(104 KB DOC)

Table S3 Type Designations and Antigenic Formulae for the 90 Serotypes of S. pneumoniae
The antigenic formulae represent arbitrary designations of cross-reactions as seen by the capsular reaction.
(72 KB DOC)

Table S4 Type and Strain Designations for the 90 Strains of S. pneumoniae Analysed (68 KB DOC)

### Accession Numbers

The EMBL Nucleotide Sequence Database (http://www.ebi.ac.uk/embl), GenBank (http://www.ncbi.nlm.nih.gov/Genbank), and DNA Data Bank of Japan (http://www.ddbj.nig.ac.jp/Welcome-e-html) accession numbers for the sequences reported in this paper for the capsular biosynthetic genes of the 90 serotypes of S. pneumoniae are CR931632–CR931722. The EMBL Nucleotide Sequence Database (http://www.ebi.ac.uk/embl) accession number for the *wzg* gene is AY336008. The Pfam domain (http://www.sanger.ac.uk/cgi-bin/Pfam) for WchA, WciI, WcjG, and WcjH is PF02397.

## Abbreviations

CPS: capsular polysaccharide
Galp: galactopyranose
GalpNAc: N-acetylgalactosamine pyranose
Glcp: glucopyranose
GlcpNAc: N-acetylglucosamine pyranose
HG[number]: homology group[number]
IS: insertion sequence
KDQNAc: 4-keto-N-acetyl-D-quinovosamine
