# Supplementary material for: Genetic Analysis of the Capsular Biosynthetic Locus from All 90 Pneumococcal Serotypes
Source: PLoS Genet. 2006 Mar 10;2(3):e31. doi: 10.1371/journal.pgen.0020031 (PMC1391919; doi:10.1371/journal.pgen.0020031)
Supplement: Supplementary file 1 [file pgen.0020031.st001.doc]

**T1**

**Supplemental Table 1: Homology groups including numbers of members and product description.**

Proteins in different homology groups are so divergent that they are highly unlikely to have diverged from a common streptococcal ancestor.

| **Homology group** | **No. members** | **product** | **gene** |
| --- | --- | --- | --- |
| 0 | 90 | CPS expression regulator Wzg* | wzg |
| 1 | 90 | Wze phosphotyrosine phosphatase Wzh* | wzh |
| 2 | 90 | Wze phosphorylation Wzd* | wzd |
| 3 | 90 | Autophosphorylating protein-tyrosine kinase Wze* | wze |
| 4 | 70 | putative IS630-Spn1 transposase | tnp |
| 5 | 65 | undecaprenylphosphate glucosephosphotransferase WchA | wchA |
| 6 | 58 | UDP-galactopyranose mutase Glf | glf |
| 7 | 41 | flippase Wzx | wzx |
| 8 | 41 | dTDP-D-glucose 4,6-dehydratase RmlB | rmlB |
| 9 | 41 | dTDP-4-keto-L-rhamnose reductase RmlD | rmlD |
| 10 | 40 | glucose-1-phosphate thymidylyltransferase RmlA | rmlA |
| 11 | 39 | dTDP-4-keto-6-deoxy-D-glucose 3,5-epimerase RmlC | rmlC |
| 12 | 33 | putative IS1670 transposase | tnp |
| 15 | 27 | putative rhamnosyl transferase WchF | wchF |
| 13 | 26 | putative IS1167 transposase | tnp |
| 16 | 24 | putative glycosyl transferase | wciB |
| 19 | 21 | putative IS1381 transposase | tnp |
| 17 | 20 | putative IS1202 transposase | tnp |
| 18 | 19 | putative acetyl transferase | wcjE |
| 20 | 18 | UDP-glucose 6-dehydrogenase Ugd | ugd |
| 21 | 16 | putative acetyl transferase | wciG |
| 23 | 15 | UDP-N-acetylglucosamine-2-epimerase MnaA | mnaA |
| 24 | 13 | putative glycosyl transferase | wcrC |
| 25 | 13 | putative glycosyl transferase | wciF |
| 26 | 12 | putative glycosyl transferase enhancer | wchJ |
| 27 | 12 | putative glycosyl transferase | wchK |
| 28 | 12 | CDP-glycerol-1-phosphate biosynthetic protein Gct | gct |
| 31 | 12 | putative IS1671 transposase | tnp |
| 47 | 12 | flippase Wzx | wzx |
| 29 | 11 | putative initial sugar transferase | wciI |
| 32 | 10 | putative glycosyl transferase | wcrD |
| 33 | 9 | putative WecB-family glycosyl transferase | wchO |
| 39 | 9 | putative IS1239 transposase | tnp |
| 40 | 9 | glycerol-2-phosphate pathway, Gtp1 | gtp1 |
| 41 | 9 | glycerol-2-phosphate pathway, Gtp2 | gtp2 |
| 42 | 9 | glycerol-2-phosphate pathway, Gtp3 | gtp3 |
| 51 | 9 | putative glycosyl transferase | wcrH |
| 35 | 8 | putative glycosyl transferase | wciJ |
| 36 | 8 | FnlA steps 1,2,3 of UDP-FucNAc synthesis | fnlA |
| 37 | 8 | FnlB step 4 of UDP-FucNAc synthesis | fnlB |
| 38 | 8 | FnlC step 5 of UDP-FucNAc synthesis | fnlC |
| 52 | 8 | flippase Wzx | wzx |
| 44 | 7 | putative glycosyl transferase | wciE |
| 45 | 7 | oligosaccharide repeat unit polymerase Wzy | wzy |
| 48 | 7 | putative acetyl transferase | wcwC |
| 49 | 7 | ribofuranose pathway gene | rbsF |
| 50 | 7 | putative initial sugar transferase | wcjG |
| 53 | 7 | putative glycerol phosphotransferase | wchX |
| 59 | 7 | putative glycosyl transferase | wciU |
| 43 | 6 | putative glycosyl transferase | wchQ |
| 55 | 6 | putative glycosyl transferase | wcxB |
| 56 | 6 | flippase Wzx | wzx |
| 57 | 6 | putative nucleotidyl transferase (arabinitol) Abp1 | abp1 |
| 58 | 6 | putative reductase (arabinitol) Abp2 | abp2 |
| 60 | 6 | putative glycosyl transferase | wciD |
| 116 | 6 | putative glycosyl transferase | wcyK |
| 34 | 5 | putative glycosyl transferase | wchN |
| 54 | 5 | putative glycosyl transferase | wcrL |
| 61 | 5 | putative initial sugar transferase | wcjH |
| 62 | 5 | putative glycosyl transferase | wciN |
| 63 | 5 | putative glycosyl transferase | wcwA |
| 64 | 5 | putative glycosyl transferase | wcwK |
| 65 | 5 | oligosaccharide repeat unit polymerase Wzy | wzy |
| 66 | 5 | putative glycosyl transferase | wcjA |
| 67 | 5 | oligosaccharide repeat unit polymerase Wzy | wzy |
| 68 | 5 | oligosaccharide repeat unit polymerase Wzy | wzy |
| 69 | 5 | putative acetyl transferase | wcwT |
| 70 | 5 | putative glycerol phosphotransferase | wcwU |
| 71 | 5 | oligosaccharide repeat unit polymerase Wzy | wzy |
| 72 | 5 | putative glycosyl transferase | wcxD |
| 73 | 5 | putative glycosyl transferase | wcxE |
| 74 | 5 | putative glycosyl transferase | wcxF |
| 75 | 5 | UDP-N-acetylmannosamine dehydrogenase MnaB | mnaB |
| 76 | 5 | putative glycosyl transferase | wchL |
| 77 | 5 | putative glycosyl transferase | wchM |
| 78 | 5 | putative glycerol phosphotransferase | wciY |
| 79 | 5 | putative LicD-family phosphotransferase | wcxG |
| 80 | 5 | putative acetyl transferase | wcyO |
| 129 | 5 | putative glycerol phosphotransferase | wcrJ |
| 46 | 4 | putative LicD-family phosphotransferase | wcrO |
| 81 | 4 | putative ribitol phosphotransferase | wciO |
| 82 | 4 | flippase Wzx | wzx |
| 83 | 4 | oligosaccharide repeat unit polymerase Wzy | wzy |
| 84 | 4 | putative glycosyl transferase | wcjB |
| 85 | 4 | flippase Wzx | wzx |
| 86 | 4 | putative glycosyl transferase | wcjC |
| 87 | 4 | putative LicD-family phosphotransferase | wcrB |
| 88 | 4 | putative acetyl transferase | wciZ |
| 89 | 4 | putative glycosyl transferase | wcrP |
| 90 | 4 | putative glycosyl transferase | wcrR |
| 91 | 4 | putative glycosyl transferase | wciV |
| 92 | 4 | putative glycosyl transferase | wciW |
| 93 | 4 | oligosaccharide repeat unit polymerase Wzy | wzy |
| 94 | 4 | hypothetical protein (pseudogene) | HG94 |
| 95 | 4 | putative LicD-family phosphotransferase | wchP |
| 96 | 4 | UDP-galacturonate 4-epimerase Gla | gla |
| 105 | 4 | putative acetyl transferase | wcxM |
| 108 | 4 | putative group II intron protein | intron |
| 111 | 4 | flippase Wzx | wzx |
| 118 | 4 | putative glycosyl transferase | wcyS |
| 149 | 4 | putative LicD-family phosphotransferase | whaI |
| 30 | 3 | putative glycosyl transferase | wciL |
| 97 | 3 | putative glycosyl transferase | wciP |
| 99 | 3 | putative glycosyl transferase | wcwI |
| 100 | 3 | putative glycosyl transferase | wcwL |
| 101 | 3 | putative glycosyl transferase | wcxU |
| 102 | 3 | putative glycosyl transferase | wcrG |
| 103 | 3 | oligosaccharide repeat unit polymerase Wzy | wzy |
| 106 | 3 | putative glycosyl transferase | wcrQ |
| 107 | 3 | putative acetyl transferase | wciX |
| 109 | 3 | putative glycosyl transferase | wchV |
| 110 | 3 | putative glycosyl transferase | wchW |
| 112 | 3 | oligosaccharide repeat unit polymerase Wzy | wzy |
| 113 | 3 | putative glycosyl transferase | wcxI |
| 114 | 3 | putative glycosyl transferase | wcxJ |
| 115 | 3 | putative LicD-family phosphotransferase | wcxK |
| 119 | 3 | putative LicD-family phosphotransferase | wcrN |
| 121 | 3 | oligosaccharide repeat unit polymerase Wzy | wzy |
| 122 | 3 | conserved hypothetical protein | wcrW |
| 123 | 3 | flippase Wzx | wzx |
| 124 | 3 | putative acetyl transferase (fragment) | wcrX |
| 125 | 3 | putative glycosyl transferase | wciC |
| 126 | 3 | oligosaccharide repeat unit polymerase Wzy | wzy |
| 127 | 3 | oligosaccharide repeat unit polymerase Wzy | wzy |
| 128 | 3 | putative glycosyl transferase | wcrI |
| 130 | 3 | putative glycosyl transferase | wcrK |
| 131 | 3 | putative nucleotidyl transferase (mannitol) Mnp1 | mnp1 |
| 132 | 3 | putative reductase (mannitol) Mnp2 | mnp2 |
| 154 | 3 | putative glycosyl transferase | wcxN |
| 155 | 3 | putative glycerol phosphotransferase | wcxP |
| 156 | 3 | putative LicD-family phosphotransferase | wcxQ |
| 170 | 3 | putative glycosyl transferase | wcyA |
| 171 | 3 | oligosaccharide repeat unit polymerase Wzy | wzy |
| 172 | 3 | putative glycosyl transferase | wcyB |
| 173 | 3 | putative glycosyl transferase | wcyC |
| 174 | 3 | putative glycosyl transferase | wcyD |
| 176 | 3 | flippase Wzx | wzx |
| 177 | 3 | conserved hypothetical protein | wcyF |
| 98 | 2 | conserved hypothetical protein | wcwD |
| 104 | 2 | putative glycosyl transferase | wcxS |
| 117 | 2 | putative glycosyl transferase | wcrM |
| 120 | 2 | putative glycosyl transferase | whaB |
| 139 | 2 | oligosaccharide repeat unit polymerase Wzy | wzy |
| 140 | 2 | putative glycosyl transferase (pseudogene) | HG140 |
| 141 | 2 | putative glycosyl transferase | wcwF |
| 142 | 2 | putative glycosyl transferase | wcwG |
| 143 | 2 | putative glycosyl transferase | wcwH |
| 144 | 2 | oligosaccharide repeat unit polymerase Wzy | wzy |
| 145 | 2 | putative acetyl transferase | wcjD |
| 146 | 2 | putative IS116-IS110-IS902-like transposase | tnp |
| 147 | 2 | putative acetyl transferase | wcwR |
| 148 | 2 | putative IS1381 transposase | tnp |
| 150 | 2 | oligosaccharide repeat unit polymerase Wzy | wzy |
| 157 | 2 | conserved hypothetical protein | wcrT |
| 158 | 2 | putative acetyl transferase | wcrU |
| 159 | 2 | putative glycosyl transferase | wcrV |
| 160 | 2 | oligosaccharide repeat unit polymerase Wzy | wzy |
| 161 | 2 | oligosaccharide repeat unit polymerase Wzy | wzy |
| 162 | 2 | hypothetical protein | wchR |
| 163 | 2 | oligosaccharide repeat unit polymerase Wzy | wzy |
| 164 | 2 | putative glycosyl transferase | wchS |
| 165 | 2 | putative glycosyl transferase | wcwV |
| 166 | 2 | oligosaccharide repeat unit polymerase Wzy | wzy |
| 167 | 2 | conserved hypothetical protein | wcwX |
| 168 | 2 | oligosaccharide repeat unit polymerase Wzy | wzy |
| 175 | 2 | putative glycosyl transferase | wcyE |
| 178 | 2 | putative acetyl transferase | wcyH |
| 179 | 2 | oligosaccharide repeat unit polymerase Wzy | wzy |
| 180 | 2 | hypothetical protein | wcyI |
| 181 | 2 | putative glycosyl transferase | wcyM |
| 182 | 2 | oligosaccharide repeat unit polymerase Wzy | wzy |
| 204 | 2 | putative glycosyl transferase | wcyN |
| 251 | 2 | oligosaccharide repeat unit polymerase Wzy | wzy |
| 133 | 1 | putative glycosyl transferase | wchG |
| 134 | 1 | putative glycosyl transferase | wchH |
| 135 | 1 | oligosaccharide repeat unit polymerase Wzy | wzy |
| 136 | 1 | putative glycosyl transferase | wchI |
| 138 | 1 | putative glycosyl transferase | whaF |
| 151 | 1 | putative LicD-family phosphotransferase | wcxR |
| 152 | 1 | oligosaccharide repeat unit polymerase Wzy | wzy |
| 153 | 1 | hypothetical protein | wcxT |
| 183 | 1 | conserved hypothetical protein | wcwY |
| 184 | 1 | putative glycosyl transferase | whaC |
| 185 | 1 | putative glycosyl transferase | whaD |
| 186 | 1 | hypothetical protein | whaE |
| 187 | 1 | putative glycosyl transferase | wcyV |
| 189 | 1 | putative glycerol phosphotransferase | whaG |
| 190 | 1 | Long repetitive protein | lrp |
| 191 | 1 | hypothetical protein (pseudogene) | HG191 |
| 192 | 1 | IS1548 transposase | tnp |
| 193 | 1 | putative glycosyl transferase | whaJ |
| 194 | 1 | glycosyl transferase | wcyT |
| 195 | 1 | putative glycosyl transferase | wcyU |
| 196 | 1 | serotype 3 capsule synthase | wchE |
| 197 | 1 | glucosephosphate uridylyltransferase | galU |
| 198 | 1 | phosphoglucomutase | pgm |
| 199 | 1 | putative glycosyl transferase | whaK |
| 200 | 1 | putative pyruvyl transferase | whaL |
| 201 | 1 | hypothetical protein | wcyP |
| 202 | 1 | putative glycosyl transferase | wcyQ |
| 203 | 1 | putative LicD-family phosphotransferase | wcyR |
| 205 | 1 | putative glycosyl transferase | whaM |
| 206 | 1 | galacturonosyl initial sugar transferase | wchB |
| 207 | 1 | putative acetyl transferase | wchC |
| 208 | 1 | putative glycosyl transferase | wchD |
| 209 | 1 | oligosaccharide repeat unit polymerase Wzy | wzy |
| 210 | 1 | flippase Wzx | wzx |
| 211 | 1 | putative glycosyl transferase | wciT |
| 212 | 1 | putative glycosyl transferase wciK | wciK |
| 213 | 1 | oligosaccharide repeat unit polymerase Wzy | wzy |
| 214 | 1 | putative pyruvyl transferase WciM | wciM |
| 215 | 1 | flippase Wzx | wzx |
| 217 | 1 | oligosaccharide repeat unit polymerase Wzy | wzy |
| 219 | 1 | putative IS1380-Spn1 transposase (fragment) | tnp |
| 220 | 1 | putative glycosyl transferase enhancer | wciQ |
| 221 | 1 | putative glycosyl transferase | wciR |
| 222 | 1 | putative glycosyl transferase WciS | wciS |
| 223 | 1 | oligosaccharide repeat unit polymerase Wzy | wzy |
| 225 | 1 | putative IS1380-Spn1 transposase | tnp |
| 230 | 1 | oligosaccharide repeat unit polymerase Wzy | wzy |
| 234 | 1 | oligosaccharide repeat unit polymerase Wzy | wzy |
| 236 | 1 | flippase Wzx | wzx |
| 237 | 1 | putative glycosyl transferase | wchU |
| 238 | 1 | oligosaccharide repeat unit polymerase Wzy | wzy |
| 240 | 1 | oligosaccharide repeat unit polymerase Wzy | wzy |
| 243 | 1 | oligosaccharide repeat unit polymerase Wzy | wzy |
| 249 | 1 | oligosaccharide repeat unit polymerase Wzy | wzy |
| 250 | 1 | flippase Wzx | wzx |
| 252 | 1 | oligosaccharide repeat unit polymerase Wzy | wzy |
| 254 | 1 | oligosaccharide repeat unit polymerase Wzy | wzy |
| 257 | 1 | oligosaccharide repeat unit polymerase Wzy | wzy |
| 258 | 1 | putative IS1672 transposase | tnp |
| 260 | 1 | oligosaccharide repeat unit polymerase Wzy | wzy |
| 264 | 1 | hypothetical protein (fragment) | HG264 |
| 266 | 1 | putative acetyl transferase (fragment) | HG266 |
| 270 | 1 | putative acetyl transferase (fragment) | HG270 |
| 273 | 1 | putative acetyl transferase (fragment) | HG273 |
| 271 | 1 | putative acetyl transferase (pseudogene) | HG271 |
| 272 | 1 | putative acetyl transferase (pseudogene) | HG272 |
| 267 | 1 | putative glycosyl transferase (fragment) | HG267 |
| 268 | 1 | putative glycosyl transferase (fragment) | HG268 |
| 269 | 1 | putative glycosyl transferase (fragment) | HG269 |
| 262 | 1 | putative LicD-family phosphotransferase (pseudogene) | HG262 |
| 263 | 1 | putative LicD-family phosphotransferase (pseudogene) | HG263 |
| 265 | 1 | UDP-galactopyranose mutase (pseudogene) | HG265 |
| 261 | 1 | undecaprenylphosphate glucosephosphotransferase WchA (fragment) | HG261 |

* Function based in part on data from other species. See Yother 2004 for recent review.
