# Supplementary material for: Genetic Analysis of the Capsular Biosynthetic Locus from All 90 Pneumococcal Serotypes
Source: PLoS Genet. 2006 Mar 10;2(3):e31. doi: 10.1371/journal.pgen.0020031 (PMC1391919; doi:10.1371/journal.pgen.0020031)
Supplement: Supplementary file 2 [file pgen.0020031.st002.doc]

**T2**

**Supplemental Table 2: Associations between initial transferases and Wzy polymerase groups.**

Proposed Wzy groupings represent a sequential numbering of homology groups and are represented on structural diagrams.

| Initial Transferase | Wzy group | HG | Polymerisation Linkage | | Serotypes |
| --- | --- | --- | --- | --- | --- |
| WchA | 1 | 45 | D-Glcp(b1-6)D-Galf | | 35A,35B,35C,42 |
|  | 4 | 68 | D-Glcp(b1-6)D-Glcp | | 11A |
|  |  |  | D-Glcp(b1-6)D-GlcpNAc | | 11F,11B,11C |
|  |  |  | NO STRUCTURE | | 11D |
|  | 5 | 83 | D-Glcp(b1-4)D-Glcp | | 9A,9V |
|  |  |  | D-Glcp(b1-4)D-GlcpNAc | | 9L,9N |
|  | 6 | 93 | D-Glcp(b1-4)D-Galp | | 18F,18A,18B,18C |
|  | 7 | 127 | D-Glcp(b1-5)D-Galp | | 33B,33D |
|  | 8 | 65 | D-Glcp(b1-4)D-Glcp | | 7B,7C,40 |
|  | 9 | 103 | D-Glcp(b1-6)D-GlcpNAc | | 14,15B,15C |
|  | 10 | 121 | NO STRUCTURE | | 41F,41A |
|  | 11 | 126 | D-Glcp(b1-5)D-Galf | | 33F,33A,37 |
|  | 12 | 139 | D-Glcp(a1-3)L-Rhap | | 6A,6B |
|  | 13 | 144 | D-Glcp(b1-3)D-GalpNAc | | 7F,7A |
|  | 14 | 150 | D-Glcp(b1-3)D-Galp | | 15F,15A |
|  | 15 | 152 | NO STRUCTURE | | 16A |
|  | 16 | 160 | D-Glcp(b1-3)D-Galp | | 17F,17A |
|  | 17 | 163 | D-Glcp(b1-4)D-ManpNAc | | 19B,19C |
|  | 18 | 166 | D-Glcp(a1-3)D-Galf | | 22F,22A |
|  | 19 | 168 | D-Glcp(b1-4)D-Galp | | 23F,23B |
|  | 21 | 251 | NO STRUCTURE | | 28A, 28F |
|  | 23 | 223 | D-Glcp(a1-4)D-Galp | | 8 |
|  | 24 | 238 | D-Glcp(b1-3)D-GlcpNAc | | 20 |
|  | 25 | 135 | D-Glcp(b1-3)L-Rhap | | 2 |
|  | 26 | 230 | D-Glcp(b1-3)D-Galf | | 13 |
|  | 27 | 234 | NO STRUCTURE | | 16F |
|  | 28 | 161 | D-Glcp(a1-3)L-Rhap | | 19A |
|  | 29 | 161 | D-Glcp(a1-2)L-Rhap | | 19F |
|  | 30 | 243 | NO STRUCTURE | | 23A |
|  | 31 | 252 | D-Glcp(a1-2)D-Galf | | 34 |
|  | 32 | 254 | NO STRUCTURE | | 36 |
|  | 34 | 112 | NO STRUCTURE | | 24F,24A,24B |
|  | 35 | 179 | D-Glcp(b1-3)D-Glcp | | 32F,32A |
|  | 40 | 240 | NO STRUCTURE | | 21 |
|  | 38 | 249 | D-Glcp(b1-3)D-GlcpNAc | | 27 |
|  | 33 | 260 | NO STRUCTURE | | 48 |
|  |  |  |  | |  |
| WciI | 3 | 71 | D-GlcpNAc(b1-4)D-ManpNAcA  D-GalpNAc(b1-4)D-ManpNAcA  NO STRUCTURE | | 12A  12F  12B,44,46 |
|  | 20 | 171 | NO STRUCTURE | | 25F,38, 25A |
|  | 36 | 257 | D-GalpNAc(b1-2)L-Rhap | | 45 |
|  | 37 | 213 | D-GalpNAc(a1-4)D-Galp | | 4 |
|  | 39 | 217 | D-Sugp(b1-4)D-Glcp | | 5 |
|  |  |  |  | |  |
| WcjG | 2 | 67 | D-Galp(b1-4)D-GalpNAc  D-Galp(a1-4)D-GalpNAc  NO STRUCTURE | | 10A  10F  10B,10C |
|  | 7 | 127 | NO STRUCTURE | | 33C |
|  | 10 | 121 | D-Galf(b1-2)L-Rhap | | 31 |
|  | 22 | 182 | NO STRUCTURE | | 47A |
|  |  |  |  | |  |
| WcjH | 1 | 45 | D-Galp(b1-6)D-Galf | | 25A,29,35F,47F |
|  | 2 | 67 | NO STRUCTURE | | 39 |
|  | 22 | 182 | NO STRUCTURE | | 43 |
|  |  |  |  | |  |
|  |  |  | |  |  |

**Non-exclusive associations:**

HG121 associates with WchA and WcjG

HG127 associates with WchA and WcjG

HG45 associates with WchA and WcjH

HG67 associates with WcjG and HG61 WcjH

HG182 associates with WcjG and WcjH
