# Supplementary material for: Genetic Analysis of the Capsular Biosynthetic Locus from All 90 Pneumococcal Serotypes
Source: PLoS Genet. 2006 Mar 10;2(3):e31. doi: 10.1371/journal.pgen.0020031 (PMC1391919; doi:10.1371/journal.pgen.0020031)
Supplement: Supplementary file 3 [file pgen.0020031.st003.doc]

**T3**

**Supplemental Table 3: Type designations and antigenic formulae for the 90 serotypes of *Streptococcus pneumoniae*.**

The antigenic formulae represent arbitrary designations of cross-reactions as seen by the capsular reaction(1-3)

| **Type** | **Antigenic formula** |
| --- | --- |
| 1 | 1a |
| 2 | 2a |
| 3 | 3a |
| 4 | 4a |
| 5 | 5a |
| 6A | 6a, 6b |
| 6B | 6a, 6c |
| 7F | 7a, 7b |
| 7A | 7a, 7b, 7c |
| 7B | 7a, 7d, 7e, 7h |
| 7C | 7a, 7d, 7f, 7g, 7h |
| 8 | 8a |
| 9A | 9a, 9c, 9d |
| 9L | 9a, 9b, 9c, 9f |
| 9N | 9a, 9b, 9e |
| 9V | 9a, 9c, 9d, 9g |
| 10F | 10a, 10b |
| 10A | 10a, 10c, 10d |
| 10B | 10a, 10b, 10c, 10d, 10e |
| 10C | 10a, 10b, 10c, 10f |
| 11F | 11a, 11b, 11e,11g |
| 11A | 11a, 11c, 11d,11e |
| 11B | 11a, 11b, 11f, 11g |
| 11C | 11a, 11b, 11c,11d, 11f |
| 11D | 11a, 11b, 11c,11e |
| 12F | 12a, 12b, 12d |
| 12A | 12a, 12c, 12d |
| 12B | 12a, 12b, 12c, 12e |
| 13 | 13a, 13b |
| 14 | 14a |
| 15F | 15a, 15b, 15c,15f |
| 15A | 15a, 15c, 15d,15g |
| 15B | 15a, 15b, 15d,15e, 15h |
| 15C | 15a, 15d, 15e |
| 16F | 16a, 16b, 11d |
| 16A | 16a, 16c |
| 17F | 17a, 17b |
| 17A | 17a, 17c |
| 18F | 18a, 18b, 18c, 18f |
| 18A | 18a, 18b, 18d |
| 18B | 18a, 18b, 18e, 18g |
| 18C | 18a, 18b, 18c, 18e |
| 19F | 19a, 19b, 19d |
| 19A | 19a, 19c, 19d |
| 19B | 19a, 19c, 19e, 7h |
| 19C | 19a, 19c, 19f, 7h |
| 20 | 20a, 20b, 7g |
| 21 | 21a |
| 22F | 22a, 22b |
| 22A | 22a, 22c |
| 23F | 23a, 23b,18b |
| 23A | 23a, 23c,15a |
| 23B | 23a, 23b, 23d |
| 24F | 24a, 24b, 24d, 7h |
| 24A | 24a, 24c, 24d |
| 24B | 24a, 24b, 24e, 7h |
| 25F | 25a, 25b |
| 25A | 25a, 25c, 38a |
| 27 | 27a, 27b |
| 28F | 28a, 28b,16b, 23d |
| 28A | 28a, 28c, 23d |
| 29 | 29a, 29b,13b |
| 31 | 31a, 20b |
| 32F | 32a, 27b |
| 32A | 32a, 32b, 27b |
| 33F | 33a, 33b, 33d |
| 33A | 33a, 33b, 33d, 20b |
| 33B | 33a, 33c, 33d, 33f |
| 33C | 33a, 33c, 33e |
| 33D | 33a, 33c, 33d, 33f, 6a |
| 34 | 34a, 34b |
| 35F | 35a, 35b, 34b |
| 35A | 35a, 35c, 20b |
| 35B | 35a, 35c, 29b |
| 35C | 35a, 35c, 20b, 42a |
| 36 | 36a, 9e |
| 37 | 37a |
| 38 | 38a, 25b |
| 39 | 39a, 10d |
| 40 | 40a, 7g, 7h |
| 41F | 41a, 41b |
| 41A | 41a |
| 42 | 42a, 20b, 35c |
| 43 | 43a, 43b |
| 44 | 44a, 44b, 12b, 12d |
| 45 | 45a |
| 46 | 46a, 12c, 44b |
| 47F | 47a, 35a, 35b |
| 47A | 47a, 43b |
| 48 | 48a |

1.	Henrichsen, J. (1995) *J Clin Microbiol* **33,** 2759-62.

2.	Lund, E. & Henrichsen, J. (1978) in *Methods in Microbiology*, eds. Bergan, T. & Norris, J. (Academic Press, London, New York, San Fransisco), pp. 241-262.

3.	Sørensen, U. B. (1993) *J Clin Microbiol* **31,** 2097-100.
