# Supplementary material for: Genetic Analysis of the Capsular Biosynthetic Locus from All 90 Pneumococcal Serotypes
Source: PLoS Genet. 2006 Mar 10;2(3):e31. doi: 10.1371/journal.pgen.0020031 (PMC1391919; doi:10.1371/journal.pgen.0020031)
Supplement: Supplementary file 4 [file pgen.0020031.st004.doc]

**T4**

**Supplemental table 4: Type and strain designations for the 90 strains of *Streptococcus pneumoniae* analysed.**

| **Type** | **Strain** |
| --- | --- |
| 1 | 519/43 |
| 2 | pn2L |
| 3 | 524/62 |
| 4 | 600/62 |
| 5 | Ambrose |
| 6A | 34351=Rodriques |
| 6B | 2616/39 |
| 7F | 554/62 |
| 7A | 2040/37 |
| 7B | Johnson |
| 7C | Sutcliff |
| 8 | 573/62 |
| 9A | Wilder |
| 9L | T9233/128/68 |
| 9N | 533/62 |
| 9V | 980/68 |
| 10F | 34355 |
| 10A | 10061/38 |
| 10B | 423/82 |
| 10C | Gro Norge |
| 11F | 34356 |
| 11A | 1813/39 |
| 11B | 8087/40 |
| 11C | Eddy nr. 53 |
| 11D | 70/86 |
| 12F | 6312 |
| 12A | 559/66 |
| 12B | Gambia 1/81 |
| 13 | 34357 |
| 14 | 34359 |
| 15F | 688/63 |
| 15A | 389/39 |
| 15B | 7904/39 |
| 15C | 553/62 |
| 16F | nr. 34361 |
| 16A | R105 |
| 17F | Rose |
| 17A | nr. 4704 |
| 18F | Gethens |
| 18A | 8609/43 |
| 18B | 1033/41 |
| 18C | 4593/40 |
| 19F | 485/61 |
| 19A | nr. 141/68 |
| 19B | nr. 4594 |
| 19C | 7588/39 |
| 20 | 34365 |
| 21 | 546/62 |
| 22F | 1772/40 |
| 22A | 3405/39 |
| 23F | Dr. Melchior |
| 23A | 1196/45 |
| 23B | 1039/41 |
| 24F | 24F L |
| 24A | 2748/40 |
| 24B | 2236/42 |
| 25F | 601/62 |
| 25A | tp 25/38, sp 65/81 |
| 27 | nr. 34371 |
| 28F | 34372 |
| 28A | 1982/45 |
| 29 | 34373 |
| 31 | nr. 34374 |
| 32F | nr. 34375 |
| 32A | 2813/41 |
| 33F | 3084/37 |
| 33A | Biehl |
| 33B | E294 |
| 33C | 7098/41 |
| 33D | CSF/79 |
| 34 | 676/74 |
| 35F | 361/39 |
| 35A | 1936/39 |
| 35B | 4356/39 |
| 35C | 7765/43 |
| 36 | 1095/39 |
| 37 | 264/3 |
| 38 | 9687/39 |
| 39 | 203/40 |
| 40 | Coleman |
| 41F | 8211/40 |
| 41A | 6803 |
| 42 | 198/71 |
| 43 | 2427/48 |
| 44 | Hammer |
| 45 | Eddy nr. 72 |
| 46 | Eddy nr. 73 |
| 47F | Eddy nr. 52 |
| 47A | L351 |
| 48 | 656/63 |
